# Effects of Engaging in Repeated Mental Imagery of Future Positive Events on Behavioural Activation in Individuals with Major Depressive Disorder

**DOI:** 10.1007/s10608-016-9776-y

**Published:** 2016-04-09

**Authors:** Fritz Renner, Julie L. Ji, Arnaud Pictet, Emily A. Holmes, Simon E. Blackwell

**Affiliations:** 1 Medical Research Council Cognition and Brain Sciences Unit, University of Cambridge, Cambridge, UK; 2 Department of Clinical Psychology, University of Geneva, Geneva, Switzerland; 3 Department for Clinical Neuroscience, Karolinska Institutet, Stockholm, Sweden; 4 Mental Health Research and Treatment Center, Department of Psychology, Ruhr-Universität Bochum, Massenbergstrasse 9-13, 44787 Bochum, Germany

**Keywords:** Behavioral activation, Mental imagery, Depression, Cognitive bias modification

## Abstract

Depression is associated with decreased engagement in behavioural activities. A wide range of activities can be promoted by simulating them via mental imagery. Mental imagery of positive events could thus provide a route to increasing adaptive behaviour in depression. The current study tested whether repeated engagement in positive mental imagery led to increases in behavioural activation in participants with depression, using data from a randomized controlled trial (Blackwell et al. in Clin Psychol Sci 3(1):91–111, 2015. doi:10.1177/2167702614560746). Participants (*N* = 150) were randomized to a 4-week positive imagery intervention or an active non-imagery control condition, completed via the internet. Behavioural activation was assessed five times up to 6 months follow-up using the Behavioural Activation for Depression Scale (BADS). While BADS scores increased over time in both groups, there was an initial greater increase in the imagery condition. Investigating mental imagery simulation of positive activities as a means to promote behavioural activation in depression could provide a fruitful line of enquiry for future research.

Major depressive disorder (MDD) is a common mood disorder accounting for the greatest disease burden among mental disorders worldwide (Whiteford et al. 2013). Although effective treatments for MDD are available, not all patients with MDD receive treatment, and a substantial proportion of those who do receive treatment do not recover. The health burden posed by depression means that it is increasingly important to find new methods to tackle it, and preferably ones that are relatively low-cost and easily accessible (Simon and Ludman 2009). One route to such treatment innovation might be to identify the mechanisms underlying currently effective treatments, and develop new, more direct, ways to target these (Holmes et al. 2014).

According to behavioural models of depression, the increased avoidance behaviour (e.g. social withdrawal) and approach deficits (e.g. engaging in fewer potentially rewarding behaviours such as exercising) associated with depression (Hopko and Mullane 2008) play a central role in its development and maintenance (Lewinsohn 1974). Consequently, these are the main targets of Behavioural Activation (BA; Martell et al. 2010), a psychological treatment that aims to help individuals with depression engage in potentially rewarding behavioural activities via functional analysis-informed activity monitoring and scheduling (Martell et al. 2010). Several Randomized Controlled Trials (RCTs) (e.g. Dimidjian et al. 2006; Moradveisi et al. 2013) and meta-analyses (e.g. Cuijpers et al. 2007; Ekers et al. 2014) have shown BA to be effective for depression. In fact, BA is now a recommended treatment option for depression in guidelines such as those of the National Institute for Health and Care Excellence (NICE; National Institute for Health and Clinical Excellence 2009).

The putative mechanism underlying the effectiveness of BA is an increased engagement in potentially rewarding behavioural activities. Such activities are diverse, and include goal-directed behaviours such as going for a walk, or meeting up with friends for dinner. However, establishing engagement in potentially rewarding behavioural activities can present challenges to both patient and therapist. Depression is characterised by lack of motivation, reduced energy, and loss of interest in and pleasure from previously enjoyable activities (anhedonia). These symptoms can make it more difficult for patients to initiate engagement in activities, and reduce the extent to which they may be experienced as rewarding. While the BA approach is specifically tailored around these difficulties, for example teaching clients to engage in scheduled activities despite their negative feelings and lack of motivation (Martell et al. 2010), identifying cognitive processes that can be used to directly address such difficulties could provide an additional and complementary route to increasing behavioural engagement. One such process could be the simulation of potentially rewarding future activities and their outcomes via mental imagery (Holmes et al. 2016).

Mental imagery is the representation and experience of sensory information without external input (Kosslyn et al. 2001; Pearson et al. 2015). A variety of studies have shown that engaging in mental imagery of a future behaviour can influence actual behaviour. For example, amongst university students who normally consumed low amounts of fruit, those allocated to a condition that involved mental imagery simulation of situations signalling the opportunity to eat fruit and their response in those situations (eating the fruit) consumed more fruit relative to those allocated to control conditions (Knäuper et al. 2011). In addition to food consumption, mental imagery has also been shown to have an impact on a wide range of behaviours, from adaptive activities such as physical exercise (Chan and Cameron 2012) and sleep (Loft and Cameron 2013), to maladaptive activities such as gambling (Whiting and Dixon 2013). It has been shown that the processing mode (mental imagery vs. verbal processing) is critical when the goal is to influence future behavioural activities. For example, participants who engaged in mental imagery of experiencing the benefits of a cable TV service were more likely to subscribe to the service, compared to participants who read (verbal processing) about the benefits of the service without receiving imagery instructions (Gregory et al. 1982). Other studies have focussed on imagery perspective (first-person vs. third-person) when studying the relation between mental imagery of specific activities and the acting out of these activities (e.g., Libby et al. 2007).

In the context of depression, mental imagery of potentially rewarding activities may be particularly useful in increasing behavioural activation (Holmes et al. 2016). Simulating possible future events or courses of action via mental imagery allows us to “pre-experience” them and their emotional consequences (Moulton and Kosslyn 2009; Schacter et al. 2008; Suddendorf and Corballis 2007), from fears (Lang 1979) to pleasures (Kavanagh et al. 2005). As such, mental imagery simulations can help individuals anticipate how emotionally rewarding, or aversive, a future event is likely to be. Depression is associated with impoverished ability to deliberately generate mental imagery of positive, but not negative, hypothetical future events (Morina et al. 2011). Training mental imagery of hypothetical future activities that result in positive outcomes may therefore provide a route to increasing adaptive behaviour in depression (Holmes et al. 2016).

Few studies have investigated mental imagery’s impact on behaviour in the context of depression. One study examining mental imagery generated in response to picture-word cue pairs in the laboratory found that dysphoric individuals performed better on a single behavioural task (number of fishes caught on a toy fishing game) when instructed to generate positive imagery in response to the picture-word cues, relative to those instructed to generate negative or both positive and negative imagery (Pictet et al. 2011). The degree to which engagement in mental imagery of positive events could increase engagement in behavioural activities in daily life in individuals with clinical levels of depression remains unknown.

The present study aimed to investigate whether a 4 week positive mental imagery intervention led to increases in behavioural activation in individuals with current major depression, using the opportunity presented by a recent randomized control trial (RCT). Blackwell et al. (2015) conducted an RCT to investigate the efficacy of an internet-delivered positive imagery intervention in reducing symptoms of depression. They recruited 150 adults with current major depression, and randomized them to complete either the positive imagery intervention (*n* = 76) or an active non-imagery control condition (*n* = 74), both delivered via the internet over a 4-week period with a 6-month follow-up.

The positive imagery intervention involved repeated generation of mental imagery in response to training stimuli that resolved positively (photo and word pairs, or audio descriptions of everyday situations), thereby aiming to train an adaptive bias to automatically imagine positive resolutions for ambiguous situations in daily life (Holmes et al. 2006, 2008). The control condition was identical to the positive imagery condition except that there was no requirement to generate imagery, and only half of the other ambiguous stimuli were resolved positively, the other half being resolved negatively. Previous experimental and preliminary clinical studies had indicated the crucial role of both the instructions to use imagery (e.g. Holmes et al. 2009; Torkan et al. 2014) and the consistent positive resolution of the ambiguous stimuli (e.g. Lang et al. 2012; Pictet et al. 2011). To investigate efficacy within an RCT framework, Blackwell et al. (2015) therefore used a control condition designed to remove both the practice in generating positive mental imagery and the training to always expect a positive resolution.

Contrary to their primary hypothesis, Blackwell et al. (2015) found no difference in reduction in symptoms of depression (as measured by the BDI-II) between the positive imagery and control conditions: participants in both conditions showed large reductions over the course of the study. However, in post hoc analyses Blackwell et al. (2015) found a greater decrease in anhedonic symptoms over the course of the intervention in the positive imagery condition compared to the control condition. They suggested that in future work, measures of specific processes or symptoms that are more closely linked to mechanisms targeted in the intervention may be more sensitive outcome measures than broad measures of disorder (such as the BDI-II).

Given the link between imagery and behaviour, and the hypothesised role of imagery in simulating and anticipating the outcome of future situations, increases in behavioural activation may present a specific outcome with close links to the positive imagery intervention. Many of the positive imagery training scenarios used in Blackwell et al. (2015) involve engaging in everyday behavioural activities, all with positive outcomes. The literature linking imagery to behaviour suggests that the imaginal engagement in activities in the positive imagery condition could lead to increased likelihood of actually engaging in those activities. Conversely, in the control condition participants did not engage in imagery, and there was not a training contingency to always expect a positive outcome from the activities presented. As the training scenarios involved a wide range of everyday activities, any impact on behaviour would likely be broad, i.e. generalized behavioural activation. To address the question of whether an imagery intervention involving imagining oneself taking part in a range of activities with positive outcomes could lead to increases in behavioural activation, we therefore analysed a secondary outcome measure of the trial, self-reported behavioural activation as assessed with the Behavioural Activation for Depression Scale (BADS; Kanter et al. 2007).

We tested the hypothesis that participants in the positive imagery condition would show greater increases in behavioural activation over the course of the study compared to those in the control condition. We further explored the effects of the positive imagery intervention on the four subscales of the BADS: activation, avoidance/rumination, work/school impairment, and social impairment. Blackwell et al. (2015) also found that the more vividly participants in the imagery condition imagined the training scenarios during the 4-week intervention (as indexed by the vividness ratings they provided after each scenario), the greater their reduction in symptoms of depression. Conversely, the difficulty ratings made by participants in the control condition showed no relationship with reduction in symptoms of depression. We therefore additionally conducted an exploratory analysis to investigate whether imagery vividness during the intervention related to increase in behavioural activation.

## Methods

Full details of participant characteristics and study procedures are described in Blackwell et al. (2015).

### Participants

Participants were 150 adults (103 female) who met DSM-IV (American Psychiatric Association 2000) criteria for a current major depressive episode assessed via a semi-structured clinical interview (SCID-I; First et al. 2002). Additionally, participants had to be fluent in written and spoken English, and able to: give informed consent, access the internet based intervention, and attend the research centre for assessment appointments. Exclusion criteria were: meeting criteria for a current psychotic or substance abuse disorder, history of mania or hypomania, having started or changed dose of antidepressant medication during the past month, currently receiving psychological therapy (including both individual or group-based psychotherapies, or counselling), or involvement in other current treatment trials.

Participants were recruited via advertisement in local media (newspapers, radio), websites (e.g. Google, Facebook), and community, university, and health settings in the local area. Initial screening was via web-based questionnaires (scoring ≥ 14, i.e. the cut-off for at least mild depression, on the Beck Depression Inventory-II; BDI-II; Beck et al. 1996) and a brief telephone interview.

Participant characteristics at baseline were: BDI-II: *M* = 30.54, *SD* 9.41; number of previous depressive episodes: 0–1: 24.7 %, 2–3: 22.0 %, ≥4: 53.3 %; currently taking an antidepressant: 42.7 %; current comorbid anxiety disorder: 54.7 %. Participants ranged in age from 18 to 65 years (*M* = 35.49, *SD* 14.05), with 94.7 % reporting their ethnicity as “White” on a checklist, and 56.7 % in paid employment, 28.0 % student, 9.3 % unemployed, 3.3 % full-time homemaker or carer, and 2.7 % retired.

Ethical approval was provided by the NRES Committee South Central - Oxford C (11/SC/0278). The trial was prospectively registered, clinicaltrials.gov identifier NCT01443234.

### Positive Imagery Condition

The positive imagery intervention comprised 12 sessions completed at home over a 4-week period. In six of the sessions, participants listened to audio recordings of descriptions of everyday situations (approximately 10 s each), and were instructed to imagine themselves in the scenarios “as if actively involved, seeing them through your own eyes.” (Holmes et al. 2006). The scenarios were initially ambiguous as to their resolution, but always ended positively. An example of a scenario is: “You have organised to go for a morning run with a friend, but wake up feeling sluggish and tired. You decide to go anyway and as you begin you start to feel exhilarated and full of energy.” In the other six sessions, participants were presented with ambiguous photos of mostly everyday scenes, paired with a caption of a few words that resolved the ambiguity in a positive way (Holmes et al. 2008; Pictet et al. 2011). Participants were instructed to generate a mental image combining the picture and words.

Each session started with reminder instructions and a practice example, followed by 64 training stimuli, arranged into eight sets of eight, with a break between each set. After each stimulus, participants rated “How vividly could you imagine the scenario described?” from 1 (*not at all vivid*) to 5 (*extremely vivid*). A session of the positive imagery training was scheduled every day in the first week, alternating between the auditory and the picture-word paradigm. Two sessions were scheduled in each of the following three weeks (one session of each paradigm).

### Non-imagery Control Condition

The intervention in the control condition was identical in all but two aspects. First, to remove the training contingency between ambiguity and positive resolution, half of the auditory training scenarios were resolved positively and half were resolved negatively. Similarly, half of the pictures had positive captions and half had negative captions. Second, to remove the mental imagery component of the training, in the auditory paradigm participants were asked to “Focus on the words and meanings” of the training scenarios, and after each one rated “How difficult was it to understand the meaning of the description?”, from 1 (*not at all difficult*) to 5 (*extremely difficult*). In the picture-word paradigm, participants were instructed to generate a sentence combining the picture and word, and after each picture-word combination rated “How difficult was it to make a sentence combining the picture with the words?” from 1 (*not at all difficult*) to 5 (*extremely difficult*).

### Measures

#### Behavioural Activation for Depression Scale (BADS; Kanter et al. 2007)

The BADS is a 25-item questionnaire originally designed to measure the kinds of behaviours hypothesised to be responsible for symptom change during Behavioural Activation treatment for depression (BA). It comprises four subscales: Activation, representing goal-directed activation and completion of scheduled activities (seven items, e.g. “*I was an active person and accomplished the goals I set out to do*”); Avoidance/Rumination, representing avoidance of negative emotional states and engaging in rumination rather than active problem-solving (eight items, e.g. “I kept trying to think of ways to solve a problem but never tried any of the solutions”); Work/School Impairment, representing inactivity with regards to school or work (five items, e.g. “I took time off of work/school/chores/responsibilities simply because I was too tired or didn’t feel like going in.”); and Social Impairment, representing social isolation (five items, e.g. *“*I was not social, even though I had opportunities to be”). Participants rate statements according to how true they were for them during the past week, on a scale from 0 (*Not at all*) to 6 (*Completely*), with 18 items reverse-scored. Higher scores indicate higher levels of behavioural activation. Validation studies indicate that the BADS has acceptable internal consistency (Cronbach’s *α* = .79) and construct validity (BADS correlation with BDI: r = -.70; Kanter et al. 2007; Kanter et al. 2009), and is sensitive to change amongst participants undergoing treatment for depression (Teismann et al. 2015).

### Procedure

Participants provided written informed consent at an initial face-to-face assessment session, after which a researcher completed the SCID interview to ascertain eligibility. Participants then returned to the research centre for the baseline assessment, during which they completed the BADS questionnaire, and were randomly allocated to one of the two conditions. The researcher then guided them through a practice session of their allocated intervention. Over the subsequent four weeks, participants completed the online intervention from home, with email/phone reminder prompts from a researcher. Participants attended a post-treatment assessment approximately four weeks after the baseline assessment, or completed the outcome measures by post/online if they were unable to attend in person. The BADS questionnaire was completed by participants online or by post at 1, 3 and 6 months follow-up after the end of the 4-week intervention. See Blackwell et al. (2015) for full details of the clinical trial procedures.

### Statistical Analyses

Change in BADS total score over the five assessment moments was modelled using mixed regression analyses in SPSS 22 following the procedures described in a recent trial of BA versus anti-depressant medication (Moradveisi et al. 2013). All available data was used in these analyses for intent-to-treat principles, including data from participants who completed the baseline assessment only. Visual inspection of average BADS scores across groups over time suggested linear and quadratic change trajectories. We therefore included linear and quadratic terms to model change over time. In these analyses, time was represented as time (in months) since baseline, starting with 0 for the initial baseline assessment. Condition was centred at +0.5 (positive imagery) and −0.5 (control), following Kraemer et al. (2002). An unstructured covariance structure was used for repeated measures. Random intercepts and slopes were not included due to convergence problems.

To test the effects of condition (positive imagery vs. control) on change in the BADS score (total and subscale scores) condition and the Time-linear × Condition and Time-quadratic × Condition interactions were added to each model. In these models, a significant Time × Condition interaction would indicate a differential change in BADS scores between the two conditions over the five assessment moments. Non-significant interaction terms were removed from the model to compute main effects. We computed the effect size (*r*) for differences between conditions from the multilevel estimates using the following formula *r* = √(*F*/(*F* + *df*)) (Moradveisi et al. 2013). Within group effect sizes were computed as the standardized mean difference of the observed values (Cohen’s *d* = (Baseline mean − mean at time i)/(√baseline variance)). Following the analytic strategy of Blackwell et al. (2015), within the positive imagery condition we repeated the mixed-model analysis with BADS total score as outcome, including the mean vividness rating across all 12 sessions as a covariate to explore the relation between imagery vividness and change in BADS scores.

## Results

### Effects of Positive Imagery Intervention on Behavioural Activation

**Fig. 1:**
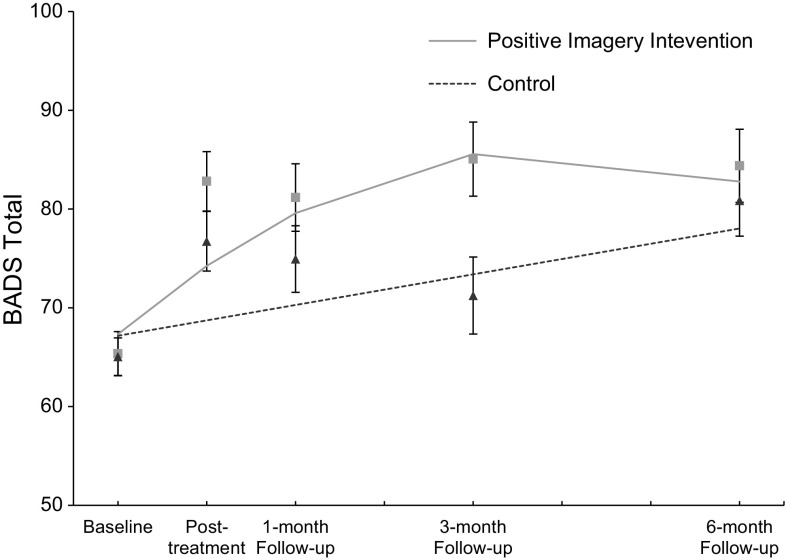
Behavioural Activation for Depression Scale (BADS) total score over five time points for both the positive mental imagery and control conditions showing mixed regression-based estimated means (*lines*) and observed means (*rectangles* for the positive imagery intervention group and *triangles* for the control group) and standard errors, indicating a significant advantage of the positive mental imagery condition. Note, the BADS total score can range from 0 to 100. For a clearer visual representation of the average scores the vertical axis in this figure starts at 50

Figure 1 shows BADS total scores over the five time points. First we tested if the positive imagery intervention had an effect on self-reported behavioural activation by testing whether the change in BADS total score over the five assessment moments differed between the positive imagery intervention and control conditions. The results of these analyses are presented in Table 1. There was a significant Condition × Time Quadratic effect, *F*(1, 133.93) = 6.86, *p* = 0.01, *r* = .22, indicating that change in the BADS total score over time differed between the two conditions. As can be seen in Fig. 1, the BADS total score increased over time in both conditions, and this increase was relatively stronger in the positive imagery condition. The increase in BADS total scores from baseline within the positive imagery intervention condition corresponded to large effect sizes at all follow-up time-points, see Table 2.

**Table 1:** Results of mixed regression analyses testing the effects of the positive mental imagery intervention on the Behavioural Activation for Depression Scale total score

	Beta	SE	DF	*t*	*p*	95 % CI
Intercept	67.25	1.44	147.50	46.79	0.00	64.41; 70.10
Time linear	4.62	1.17	134.24	3.94	0.00	2.31; 6.94
Time quadratic	−0.39	0.15	133.93	−2.63	0.01	−0.69; −0.10
Condition	0.17	2.87	147.50	0.06	0.95	−5.52; 5.85
Condition × time linear	6.13	2.34	134.24	2.62	0.01	1.50; 10.77
Condition × time quadratic	−0.78	0.30	133.93	−2.62	0.01	−1.37; −0.19

**Table 2:** Within group effect sizes (Cohen’s *d*) of change in Behavioural Activation for Depression Scale (BADS) total scores from baseline to post-treatment, 1, 3, and 6 month follow-up

	Cohen’s *d*			
	Post-treatment	1 month FU	3 month FU	6 month FU
*BADS Total Score*				
Positive imagery condition	0.89	0.81	1.01	0.97
Control condition	0.71	0.60	0.38	0.96
*BADS Subscales*				
Activation				
Positive imagery condition	0.54	0.38	0.37	0.38
Control condition	0.14	0.31	0.11	0.39
Avoidance/rumination				
Positive imagery condition	0.74	0.86	0.91	0.95
Control condition	0.55	0.37	0.26	0.81
Work/school impairment				
Positive imagery condition	0.52	0.35	0.65	0.56
Control condition	0.46	0.51	0.31	0.45
Social impairment				
Positive imagery condition	0.58	0.55	0.77	0.71
Control condition	0.48	0.15	0.18	0.53

Cohen’s *d* = (Baseline mean − mean at time i)/(√baseline variance)

### Effects of Positive Imagery Intervention on BADS Subscales

**Fig. 2:**
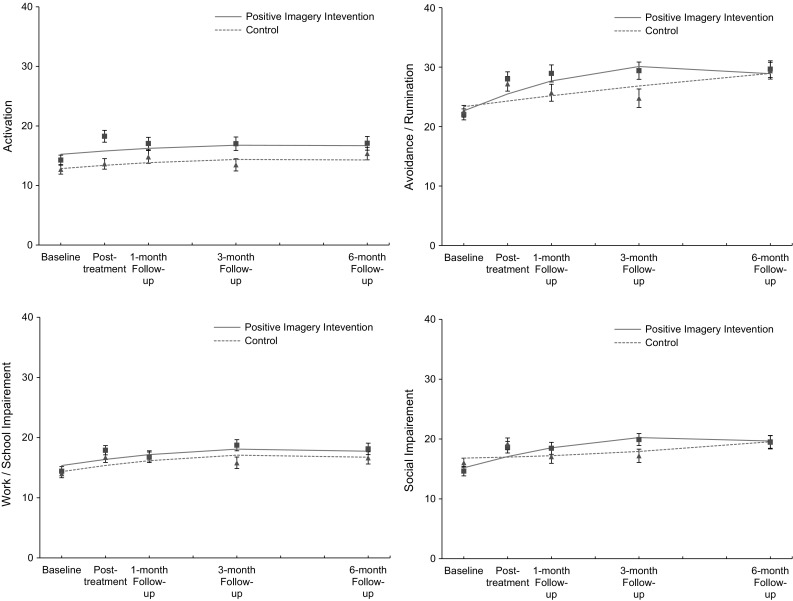
Behavioural Activation for Depression Scale (BADS) subscales over five time points for both the positive mental imagery and control conditions showing mixed regression-based estimated means (*lines*) and observed means (*rectangles* for the positive imagery intervention group and *triangles* for the control group) and standard errors, showing the significant advantage of the positive mental imagery condition on the Avoidance/Rumination and Social Impairment subscales

Next we tested effects of the positive imagery intervention on the four BADS subscales: Activation, Avoidance/Rumination, Work/School Impairment, Social Impairment. The results of these analyses are displayed in Fig. 2, and within-group effect sizes are shown in Table 2.

In the model testing intervention effects on the *Activation* subscale, the Condition × Time interactions were not significant: Condition × Time quadratic: *F*(1, 137.13) = 1.32, *p* = 0.25, *r* = 0.10; Linear interaction (after removing non-significant quadratic term: *F*(1, 129.59) = 0.22, *p* = 0.64, *r* = 0.04, indicating that change in *Activation* ratings over time did not differ between the two conditions. After removing the non-significant interaction terms from the model, there was a significant main effect of condition, *F*(1, 146.22) = 5.93, *p* = 0.02, *r* = 0.20, but not time linear, *F*(1, 140.11) = 2.49, *p* = 0.12, *r* = 0.13 indicating that participants in the positive imagery condition generally reported higher *Activation* ratings than those in the control condition throughout the study.

In the model testing intervention effects on the *Avoidance/Rumination* subscale there was a significant Condition × Time Quadratic interaction, *F*(1, 130.96) = 6.73, *p* = 0.01, *r* = 0.22 indicating that change in *Avoidance/Rumination* scores over time differed between the two conditions. As can be seen from Fig. 2, the increase on this scale was initially stronger in the positive imagery condition compared to the control condition, and then levelled out such that scores between the two conditions were comparable at the 6-month follow-up assessment.

In the model testing intervention effects on the *Work/School Impairment* subscale, the Condition × Time Quadratic interaction was not significant, *F*(1, 136.18) = 1.59, *p* = 0.21, *r* = 0.11 and neither was the Condition × Time Linear interaction, *F*(1, 129.99) = 1.60, *p* = 0.21, *r* = 0.11, indicating that scores on this subscale did not change differentially between the two conditions. After removing the non-significant interaction terms from the model, significant main effects emerged for time linear, *F*(1, 138.79) = 12.76, *p* < 0.001, *r* = 0.29, and time quadratic, *F*(1, 137.73) = 8.26, *p* < 0.01, *r* = 0.29, indicating that scores on this subscale decreased over time. The main effect of condition was not significant, *F*(1, 146.25) = 1.31, *p* = 0.26, *r* = 0.09, indicating that participants in the two conditions generally did not differ on their ratings of this subscale.

In the model testing intervention effect on the *Social Impairment* subscale of the BADS, there was a significant Condition × Time Quadratic interaction, *F*(1, 132.90) = 8.53, *p* < 0.01, *r* = 0.25, indicating that change in *Social Impairment* scores over time differed between the two condition. As can be seen from Fig. 2, scores on this subscale increased initially in the positive imagery condition and then remained roughly stable such that scores between conditions were comparable at the final follow-up assessment.

### Imagery Vividness and Increase in Behavioural Activation

We investigated whether the extent to which participants showed an increase in behavioural activation was related to how successfully they were able to engage with a putative crucial component of the positive imagery intervention—the generation of vivid imagery. A significant Time × Vividness interaction, *F*(1, 69.25) = 9.63, *p* = .003 and significant Quadratic Time × Vividness interaction, *F*(1, 68.94) = 6.16, *p* = .016 indicated that the more vividly participants in the positive imagery condition imagined the training scenarios, the greater the increase in their behavioural activation over the course of the study. The mean vividness rating did not correlate with baseline depression severity, *r*(75) = .16, *p* = .17, and when baseline depression severity was included in the mixed model analysis above the Time × Vividness and Quadratic Time × Vividness interactions remained significant, suggesting that the relationship between mean vividness rating and change in BADS score over time was not simply a reflection of baseline depression severity.

While Blackwell et al. (2015) found that vividness ratings for the first block of the practice session predicted subsequent decrease in symptoms of depression, we did not find that vividness ratings for the first block of the practice session predicted subsequent increase in behavioural activation (Time × Vividness interaction, *F*(1, 66.91) = 1.45, *p* = .23; Quadratic Time × Vividness interaction, *F*(1, 66.62) = 0.72, *p* = .40).

Within the control condition, there was no evidence for a relationship between difficulty ratings and increase in BADS, as indicated by a nonsignificant Time × Difficulty interaction, F(1, 60.98) = 0.61, *p* = .44, and a nonsignificant Quadratic Time × Difficulty interaction, F(1, 60.60) = 0.23, *p* = .63 in an equivalent mixed model analysis that included the mean difficulty rating as a covariate.

## Discussion

The current study provides initial evidence that repeated practice in generating positive mental imagery can boost self-reported behavioural activation in daily life for individuals with depression. The training scenarios participants imagined included a wide range of specific goal-directed daily situations, such as meeting a friend, or walking in the park, which always ended in a positive resolution. Why might the act of repeatedly simulating oneself engaging in these activities influence daily behaviour?

Contemporary theorists view mental imagery as a core component of the “prospective brain”, the primary function of which is to predict and plan for the future via simulations that enable us to “pre-experience” hypothetical events (Moulton and Kosslyn 2009; Schacter et al. 2008; Suddendorf and Corballis 2007). Decades of research have shown that mental imagery has the capacity to evoke emotional responses at subjective, physiological and neural levels (c.f. Ji et al. in press). Compared to verbal thinking, mental imagery is not only experienced as emotionally more powerful (Holmes and Mathews 2005), but also more real and more easily confused with real experience in memory (Mathews et al. 2013). It is possible that repeated imaginal engagement in daily activities during the training, particularly given their positive outcomes, increased participants’ expectation of finding similar activities in their day to day life rewarding, and therefore increased the likelihood of them engaging in this behaviour. However, this remains a hypothesis, and future research should test the mechanisms through which mental imagery might lead to increased engagement in behavioural activities.

The present study also evaluated the effects of the positive imagery intervention on the subscales of the BADS. Improvements on the Avoidance/Rumination subscale and the Social impairment subscale were greater in the positive imagery condition compared to the control condition, whereas improvements on the Activation subscale and on the Work/School Impairment subscales did not differ between the two conditions. Although there was no difference in improvements on the Activation subscale between conditions, participants in the positive imagery condition showed higher scores on this subscale than those in the control condition across the study assessments, with this difference appearing most pronounced at the first post-treatment assessment (Fig. 2). The finding that participants in the positive imagery condition showed greater improvements on the Social Impairments subscale of the BADS is particularly intriguing, given that many scenarios in the positive imagery intervention involved social situations. The degree to which the specific content of imagined scenarios relates to engagement in specific behaviours should be tested in future studies.

We also explored the relation between imagery vividness and changes in behavioural activation and found that the more vividly participants in the positive imagery condition reported imagining the training scenarios during the 4-week intervention, the more their behavioural activation increased over the course of the study. This mirrors the findings reported by Blackwell et al. (2015), that higher vividness ratings in the positive imagery condition were associated with greater reductions in symptoms of depression. Increase in BADS score could not be predicted from vividness ratings in the first block of the practice session (while Blackwell et al. 2015 found that these vividness ratings did predict reduction in symptoms of depression), potentially suggesting that initial imagery vividness is less important for subsequent behavioural activation than it is for reduction in symptoms of depression. Overall, the results of these exploratory analyses are consistent with the hypothesis that successful engagement with mental imagery is an important aspect for the beneficial effects of the intervention (whether decrease in depression symptoms or increase in behavioural activation). The specific role of positive imagery vividness in decreasing depressive symptoms and/or increasing adaptive behavioural outcomes warrants further investigation in future research.

The present findings fit within a broader literature that calls for enhancing positive aspects of cognitive-affective processing in disorders such as depression, rather than only focussing on reducing negative aspects (MacLeod 2012; MacLeod and Moore 2000). This may be particularly beneficial in trying to enhance the ability to anticipate or experience reward from activities in depression (Dunn 2012), which has clear links to increasing adaptive (reward-seeking) behaviour. The positive affect generated within the positive imagery sessions themselves may also contribute to their beneficial impact on behaviour. Positive emotions, the topic of this special issue, can have an impact on a range of information-processing biases (Fredrickson and Branigana 2005; Sanchez and Vazquez 2014), which could themselves have a downstream impact on behaviour.

### Implications

The findings of this study provide initial evidence suggesting that mental imagery could be used to help people with depression increase engagement in potentially rewarding behavioural activities. While the findings do not suggest a clear superiority of the imagery intervention on self-reported behavioural activation across the whole of the study period (Fig. 1), they suggest that positive mental imagery as a means to boost behavioural activation in depression could be a potentially fruitful line of enquiry for future research. Behavioural Activation (BA) theory postulates that behavioural withdrawal symptoms in depression function as a coping strategy (Martell et al. 2001). However, in avoiding environments low in positive reinforcement or high in aversive control, opportunities for exposure to positive reinforcement for adaptive behaviours are also reduced, thereby maintaining depressed mood (Martell et al. 2001). BA treatment for depression therefore aims to increase opportunities for non-depressive behaviours via engaging in potentially rewarding daily activities (Lejuez et al. 2001).

Mental imagery simulations of such potentially pleasurable daily activities could be used to facilitate the BA treatment process via two ways. First, imagining oneself engaging in behaviours can increase the intention and motivation to engage in those behaviours, such as social behaviours (Crisp et al. 2010) and health behaviours (Rennie et al. 2014a, b). Second, mental imagery enables the simulation of real life experiences, allowing the individual to not only anticipate what might happen, but also how it might feel to experience such events (Kavanagh et al. 2005; Lang 1979; Moulton and Kosslyn 2009; Schacter et al. 2008). In addition, previous research has shown that thinking about positive or negative scenarios using mental imagery elicits higher levels of positive or negative emotions than verbal elaboration of the same information (Holmes et al. 2006, 2008, 2009). As such, mental imagery simulation of pleasurable daily activities could potentially facilitate BA treatment of depression via increased intention and motivation to engage in such activities, and via increased anticipation of pleasure from engaging in them. It has been shown, for example, that in a sample of older adults with depression the most commonly scheduled activities in BA are physical activities/exercising (Riebe et al. 2012). Mental imagery training of engaging in physical activities has been shown to increase engagement in physical activities in inactive individuals (Chan et al. 2012). Similar imagery procedures could therefore be used to help individuals with depression to engage in physical activities or other scheduled activities. However, further laboratory-based research is needed to determine the specific role that mental imagery might play in increasing engagement in potentially rewarding behaviours in depression before testing its clinical application.

The findings of the present analyses provide preliminary support for this line of investigation and also highlight the potential importance of engagement with mental imagery (vividness). Experimental studies exploring how mental imagery of behavioural activities leads to increased engagement of those activities would not only be relevant for potential clinical applications, such as in BA for depression, but also for a range of other adaptive behaviours in clinical and healthy populations, such as engagement in physical exercise.

In the trial overall, there was no difference between the two conditions in terms of reduction in symptoms of depression as a whole over the course of the study, and thus any changes in behavioural activation observed were not large enough to have a downstream effect on symptoms of depression as measured with the BDI-II. Blackwell et al. (2015) suggest that specific aspects of depression that relate more closely to the processes targeted by the intervention (in their case, anhedonia) may be more suitable as targets for cognitive training interventions, as opposed to broader global measures such as symptoms of depression as a whole (for a discussion relating to the wider treatment development literature, see Reardon 2014). The findings of the current study are consistent with this suggestion. If the positive imagery intervention in this format does not have an impact on the broader depression symptom constellation, but on specific aspects of functioning or symptoms, it may be best used as an adjunct to other treatments, and perhaps refining the paradigm to target specific processes (e.g. behaviour) more powerfully may lead to a larger effect that does in fact have a downstream impact on depression symptoms.

Another interesting issue to consider is the increase in BADS scores also seen in the control condition. It is possible that simple exposure to those activity-related scenarios that resolved positively, or reflection on the scenario content between sessions, led to increases in activity (see Blackwell et al. 2015 for similar discussion in relation to depression outcomes). The increase in BADS scores could also reflect the passage of time, or a secondary effect of the decrease in depression symptoms (which showed large decreases over the course of the study; see Blackwell et al. 2015). However, participants in both conditions showed equally large improvements in depression symptoms (see Blackwell et al. 2015), with no indication in the intention-to-treat analyses of any difference between the two conditions (largest between-group effect size, Cohen’s *d*, of 0.07). Thus, finding a greater improvement in BADS scores in the positive imagery condition suggests that its impact on BADS scores was not simply due to improvement in depression, and highlights other potential mechanisms (such as imagery-behaviour links) that will be important to investigate in future studies.

### Limitations and Future Directions

The present results must be interpreted in the context of the trial in which the data was collected. First, the current study involves exploratory analyses of a secondary outcome of the original trial. While the results provide an indication of the potential of positive imagery as a means to promote behavioural activation in depression, this now needs demonstrating in a study specifically designed to test this possibility. Ideally, such studies should also aim to determine the extent to which improvements in behavioural activation drive improvements in other outcomes such as depressive symptoms and vice versa, for example by establishing the temporal sequence of the changes during the intervention.

Second, the effects of the positive imagery intervention on behavioural activation compared to the control condition, while statistically significant, are modest in size. The standard errors of the BADS total score for the two groups overlap at several time points, suggesting that the observed means for the BADS total score did not differ between the two groups at these specific time points during the study. Visual inspection of Fig. 1 suggests that between-group differences only became apparent at the 3-month follow-up assessment, and had diminished again by the 6-month follow-up assessment, with similar patterns observed for the Avoidance/Rumination and Social Impairment subscales. Given the relatively low intensity and time-limited nature of the training program, long term effects (i.e. at 6 month follow-up) may not be expected. The time course of effects and the question of how to best maintain improvements in behavioural activation over time constitute an important issue for future research. The positive imagery intervention was designed to target negative interpretation bias in depression rather than coming from a behavioural activation framework per se; future research could tailor a positive imagery intervention around increasing engagement in potentially rewarding behavioural activities in line with applied behavioural activation principles (Martell et al. 2010).

Third, as the positive imagery and control conditions differed in two aspects (use of imagery, and whether the ambiguous training stimuli were consistently resolved positively), it is unclear whether the superiority of the positive imagery over the control condition in increasing behavioural activation was specifically due to either one or both of these components.

Fourth, we assessed self-reported behavioural activation using the behavioural activation for depression scale (BADS), a measure that was designed to assess the hypothesised change processes in BA for depression. Future studies using similar procedures involving positive imagery of future behavioural activities should aim to more closely match the outcome assessment to the content of the imagery scenarios. Future research should also seek to include objective behavioural measures (e.g. using activity tracking) in addition to self-report measures which might give a more objective account of the specific behavioural activities in which participants engaged.

### Conclusions

This study provides preliminary evidence that engaging in positive mental imagery over a four week internet intervention can boost engagement in potentially rewarding behavioural activities in individuals with depression. These findings can inform future studies developing imagery interventions to target behavioural outcomes in non-clinical as well as clinical settings. The findings also add to the broader literature showing that mental imagery of specific behaviours can increase engagement in these behaviours. Future studies should evaluate the effects of positive mental imagery training programs that target potentially rewarding behavioural activities on behavioural activation, combining basic research in mental imagery with its clinical application.

## References

[CR1] American Psychiatric Association. (2000). *Diagnostic and Statistical Manual of Mental Disorders. Text revision* (4th ed.). Washington D.C.: American Psychiatric Association.

[CR2] Beck AT, Steer RA, Brown GK (1996). Manual for the Beck Depression Inventory-II.

[CR3] Blackwell SE, Browning M, Mathews A, Pictet A, Welch J, Davies J, Holmes EA (2015). Positive imagery-based cognitive bias modification as a web-based treatment tool for depressed adults: a randomized controlled trial. Clinical Psychological Science.

[CR4] Chan CKY, Cameron LD (2012). Promoting physical activity with goal-oriented mental imagery: a randomized controlled trial. Journal of Behavioral Medicine.

[CR5] Crisp Richard J, Husnu Senel, Meleady Rose, Stathi Sofia, Turner Rhiannon N (2010). From imagery to intention: A dual route model of imagined contact effects. European Review of Social Psychology.

[CR6] Cuijpers P, Van Straten A, Warmerdam E (2007). Behavioral activation treatments of depression: A meta-analysis. Clinical Psychology Review.

[CR7] Dimidjian S, Hollon SD, Dobson KS, Schmaling KB, Kohlenber RJ, Addis ME, Jacobson NS (2006). Randomized trial of behavioural activation, cognitive therapy, and antidepressant medication in the acute treatment of adults with major depression. Journal of Consulting and Clinical Psychology.

[CR8] Dunn BD (2012). Helping depressed clients reconnect to positive emotional experience: Current insights and future directions. Clinical Psychology and Psychotherapy.

[CR9] Ekers D, Webster L, Van Straten A, Cuijpers P, Richards D, Gilbody S (2014). Behavioural activation for depression; an update of meta-analysis of effectiveness and sub group analysis. PLoS ONE.

[CR10] First MB, Spitzer RL, Gibbon M, Williams JBW (2002). Structured clinical interview for DSM-IV-TR Axis I Disorders, Research Version, Patient Edition (SCID-I/P).

[CR11] Fredrickson BL, Branigana C (2005). Positive emotions broaden the scope of attention and thought-action repertoires. Cognition and Emotion.

[CR12] Gregory, W. L., Cialdini, R. B., & Carpenter, K. M. (1982). Self-relevant scenarios as mediators of likelihood estimates and compliance: Does imagining make it so. *Journal of Personality and Social Psychology, 43*(1), 89–99. doi:10.1037/0022-3514.43.1.89.

[CR13] Holmes EA, Blackwell SE, Burnett Heyes S, Renner F, Raes F (2016). Mental imagery in depression: Phenomenology, potential mechanisms, and treatment implications. Annual Review of Clinical Psychology.

[CR14] Holmes EA, Craske MG, Graybiel AM (2014). Psychological treatments: A call for mental-health science. Clinicians and neuroscientists must work together to understand and improve psychological treatments. Nature.

[CR15] Holmes EA, Lang TJ, Shah DM (2009). Developing interpretation bias modification as a ‘cognitive vaccine’ for depressed mood: Imagining positive events makes you feel better than thinking about them verbally. Journal of Abnormal Psychology.

[CR16] Holmes EA, Mathews A (2005). Mental imagery and emotion: A special relationship?. Emotion.

[CR17] Holmes EA, Mathews A, Dalgleish T, Mackintosh B (2006). Positive interpretation training: Effects of mental imagery versus verbal training on positive mood. Behavior Therapy.

[CR18] Holmes EA, Mathews A, Mackintosh B, Dalgleish T (2008). The causal effect of mental imagery on emotion assessed using picture–word cues. Emotion.

[CR19] Hopko DR, Mullane CM (2008). Exploring the relation of depression and overt behavior with daily diaries. Behaviour Research and Therapy.

[CR20] Ji, J. L., Burnett Heyes, S., MacLeod, C., & Holmes, E. A. (in press). Emotional mental imagery as simulation of reality: fear and beyond. A tribute to Peter Lang. *Behavior Therapy*. doi:10.1016/j.beth.2015.11.004 10.1016/j.beth.2015.11.004 PMC5112008 27816082

[CR21] Kanter JW, Mulick PS, Busch AM, Berlin KS, Martell CR (2007). The Behavioral Activation for Depression Scale (BADS): Psychometric properties and factor structure. Journal of Psychopathology and Behavioral Assessment.

[CR22] Kanter JW, Rusch LC, Busch AM, Sedivy SK (2009). Validation of the Behavioral Activation for Depression Scale (BADS) in a community sample with elevated depressive symptoms. Journal of Psychopathology and Behavioral Assessment.

[CR23] Kavanagh DJ, Andrade J, May J (2005). Imaginary relish and exquisite torture: The elaborated intrusion theory of desire. Psychological Review.

[CR24] Knäuper B, McCollam A, Rosen-Brown A, Lacaille J, Kelso E, Roseman M (2011). Fruitful plans: Adding targeted mental imagery to implementation intentions increases fruit consumption. Psychol Health.

[CR25] Kosslyn SM, Ganis G, Thompson WL (2001). Neural foundations of imagery. Nature Reviews Neuroscience.

[CR26] Kraemer HC, Wilson GT, Fairburn CG, Agras WS (2002). Mediators and moderators of treatment effects in randomized clinical trials. Archives of General Psychiatry.

[CR27] Lang PJ (1979). A bio-informational theory of emotional imagery. Psychophysiology.

[CR28] Lang TJ, Blackwell SE, Harmer CJ, Davison P, Holmes EA (2012). Cognitive bias modification using mental imagery for depression: Developing a novel computerized intervention to change negative thinking styles. European Journal of Personality.

[CR29] Lejuez CW, Hopko DR, LePage JP, Hopko SD, McNeil DW (2001). A brief behavioral activation treatment for depression. Cognitive and Behavioral Practice.

[CR30] Lewinsohn PM (1974). A behavioral approach to depression.

[CR31] Libby LK, Shaeffer EM, Eibach RP, Slemmer JA (2007). Picture yourself at the polls-visual perspective in mental imagery affects self-perception and behavior. Psychological Science.

[CR32] Loft MH, Cameron LD (2013). Using mental imagery to deliver self-regulation techniques to improve sleep behaviors. Annals of Behavioral Medicine.

[CR33] MacLeod AK (2012). Well-being, positivity and mental health: an introduction to the special issue. Clinical Psychology and Psychotherapy.

[CR34] MacLeod AK, Moore R (2000). Positive thinking revisited: Positive cognitions, well-being and mental health. Clinical Psychology and Psychotherapy.

[CR35] Martell CR, Addis ME, Jacobson NS (2001). Depression in context: Strategies for guided action.

[CR36] Martell CR, Dimidjian S, Herman-Dunn R (2010). Behavioral activation for depression: A clinician’s guide.

[CR37] Mathews A, Ridgeway V, Holmes EA (2013). Feels like the real thing: Imagery is both more realistic and emotional than verbal thought. Cognition and Emotion.

[CR38] Moradveisi L, Huibers MJH, Renner F, Arasteh M, Arntz A (2013). Behavioural activation v. antidepressant medication for treating depression in Iran: randomised trial. The British Journal of Psychiatry.

[CR39] Morina N, Deeprose C, Pusowski C, Schmid M, Holmes EA (2011). Prospective mental imagery in patients with major depressive disorder or anxiety disorders. Journal of Anxiety Disorders.

[CR40] Moulton ST, Kosslyn SM (2009). Imagining predictions: Mental imagery as mental emulation. Philosophical Transactions of The Royal Society B: Biological Sciences.

[CR41] National Institute for Health and Clinical Excellence (2009). Depression: The treatment and management of depression in adults (update).

[CR42] Pearson J, Naselaris T, Holmes EA, Kosslyn SM (2015). Mental imagery: Functional mechanisms and clinical applications. Trends in Cognitive Sciences.

[CR43] Pictet A, Coughtrey AE, Mathews A, Holmes EA (2011). Fishing for happiness: The effects of positive imagery on interpretation bias and a behavioral task. Behaviour Research and Therapy.

[CR44] Reardon S (2014). NIH rethinks psychiatry trials. Nature.

[CR45] Rennie L, Harris PR, Webb TL (2014). The impact of perspective in visualizing health-related behaviors: First-person perspective increases motivation to adopt health-related behaviors. Journal of Applied Social Psychology.

[CR46] Rennie L, Uskul AK, Adams C, Appleton K (2014). Visualisation for increasing health intentions: enhanced effects following a health message and when using a first-person perspective. Psychol Health.

[CR47] Riebe G, Fan MY, Unutzer J, Vannoy S (2012). Activity scheduling as a core component of effective care management for late-life depression. International Journal of Geriatric Psychiatry.

[CR48] Sanchez A, Vazquez C (2014). Looking at the eyes of happiness: Positive emotions mediate the influence of life satisfaction on attention to happy faces. The Journal of Positive Psychology.

[CR49] Schacter DL, Addis DR, Buckner RL (2008). Episodic simulation of future events concepts, data, and applications. New York Academy of Sciences.

[CR50] Simon, G. E., & Ludman, E. J. (2009). It’s time for disruptive innovation in psychotherapy. *Lancet, 374*(9690), 594–595. Retrieved from <Go to ISI>://000269222600007 10.1016/S0140-6736(09)61415-X 19699995

[CR51] Suddendorf T, Corballis MC (2007). The evolution of foresight: What is mental time travel, and is it unique to humans?. Behavioral and Brain Sciences.

[CR52] Teismann T, Ertle A, Furka N, Willutzki U, Hoyer J (2015). The German Version of the Behavioral Activation for Depression Scale (BADS): A psychometric and clinical investigation. Clinical Psychology and Psychotherapy.

[CR53] Torkan H, Blackwell SE, Holmes EA, Kalantari M, Neshat-Doost HT, Maroufi M, Talebi H (2014). Positive imagery cognitive bias modification in treatment-seeking patients with major depression in Iran: A pilot study. Cognitive Therapy and Research.

[CR54] Whiteford HA, Degenhardt L, Rehm J, Baxter A, Ferrari AJ, Erskine HE, Vos T (2013). Global burden of disease attributable to mental and substance use disorders: findings from the Global Burden of Disease Study 2010. The Lancet.

[CR55] Whiting SW, Dixon MR (2013). Effects of mental imagery on gambling behavior. Journal of Gambling Studies.

